# The effectiveness of participation in group sports-recreational activities on the quality of life of patients with schizophrenia

**DOI:** 10.1007/s44192-026-00533-1

**Published:** 2026-06-30

**Authors:** Rasool Abedanzadeh, Maryam Sepehrikia, Ayoub Hashemi

**Affiliations:** 1 Department of Motor Behavior and Sport Psychology, Faculty of Sport Sciences, Shahid Chaman University of Ahvaz, Ahvaz, Iran; 2 Department of Sport Science, Faculty of Literature and Human Sciences, Yasouj University, Yasouj, Iran

**Keywords:** Physical health, Psychological health, Sports, Schizophrenia

## Abstract

**Background and objective:**

Physical activity is an effective non-pharmacological approach to enhance the well-being of individuals with schizophrenia. This study examined the impact of group sports-recreational activities on the quality of life of patients with schizophrenia.

**Methodology:**

The statistical population of this semi-experimental study with a pretest–posttest design included 75 men with schizophrenia (mean age = 53.77 ± 4.74 years) residing in the neuropsychological rehabilitation center of Susa city in southern Iran in 2024. Sixty participants were randomly selected and assigned to either an experimental group (n = 30) or a control group (n = 30). The experimental group engaged in group sports-recreational activities for 12 weeks (three sessions per week, 20 min per session). Data were collected using demographic questionnaire and the World Health Organization’s Quality of Life questionnaire, covering four subscales: physical health, psychological health, social relationships, and environmental health.

**Findings:**

ANCOVA results, controlling for pretest scores, revealed significant differences between the groups in overall quality of life (F = 45.42, *P* = 0.001, η^2^ = 0.61). Significant improvements were also observed in physical health (F = 12.26, *P* = 0.002, η^2^ = 0.30), psychological health (F = 31.22, *P* = 0.001, η^2^ = 0.52), social relationships (F = 12.20, *P* = 0.002, η^2^ = 0.37), and environmental health (F = 10.80, *P* = 0.003, η^2^ = 0.30) for the experimental group compared to the control group.

**Conclusion:**

Group sports-recreational activities significantly improve the quality of life of patients with schizophrenia and should be included in rehabilitation programs.

## Introduction

Mental disorders are increasingly recognized as one of the most critical health challenges worldwide, contributing substantially to the global burden of disease [10, 27]. Among these, depression, bipolar disorder, schizophrenia, and cognitive impairments account for the highest proportion, with their prevalence showing a consistent upward trend. Schizophrenia, in particular, represents one of the most complex and costly psychiatric conditions, posing serious individual, social, and economic challenges for healthcare systems [31, 36]. The global prevalence of schizophrenia is estimated to range from 1 to 1.9%, with nearly two-thirds of affected individuals requiring hospitalization, while only half receive adequate treatment [5]. Schizophrenia is a chronic and severe psychiatric disorder characterized by disturbances in thought, perception, emotion, and behavior. Its symptoms are generally categorized into positive, negative, and cognitive domains. Positive symptoms include delusions, hallucinations, and disorganized thought or behavior, whereas negative symptoms encompass reduced speech, emotional blunting, avolition, and social withdrawal [2, 5]. The disorder typically manifests in early adulthood, leading to significant impairments in social and occupational functioning. Schizophrenia ranks as the fifth leading cause of years lived with disability among individuals aged 15–44 [26].

Quality of life (QoL) is considered a key outcome measure in both physical and mental health research. It reflects an individual’s perception of well-being, life satisfaction, and ability to function in daily life [10]. According to the World Health Organization, QoL is defined as an individual’s perception of their position in life within the context of their cultural values, goals, expectations, and standards [26]. Patients with schizophrenia experience poorer physical health compared to the general population [13]. Sedentary lifestyle patterns, unhealthy diets, smoking, alcohol consumption, and long-term antipsychotic medication use increase the risk of comorbid conditions such as hypertension, type 2 diabetes, and cardiovascular disease [19, 23]. Antipsychotic-induced metabolic side effects, including weight gain, further exacerbate physical health problems and diminish quality of life. In schizophrenia, QoL is influenced by psychiatric symptoms, sociodemographic variables, employment status, living conditions, and physical factors such as high body mass index [43]. Men often exhibit earlier onset, more severe negative symptoms, and lower social functioning compared to women [5]. Premature mortality among patients with schizophrenia is largely attributed to cardiovascular disease, resulting in significantly reduced life expectancy [7]. Therefore, enhancing quality of life has become a primary objective in psychiatric care and policy development [26]. In recent decades, attention has increasingly shifted from pharmacological to non-pharmacological interventions, particularly physical and recreational activities [19, 25]. Physical activity has been widely recognized as an effective means of promoting physical and psychological well-being and preventing disease [20]. Empirical evidence indicates that exercise programs—including walking, resistance training, and group-based activities—can improve social interaction, self-efficacy, and mental health in individuals with schizophrenia [14].

Because motivation and adherence to exercise programs are often low among individuals with chronic mental disorders, integrating elements of enjoyment, creativity, and social engagement into activity design is crucial to sustaining participation [19, 27].Motivational enhancement strategies and adherence-focused approaches can significantly improve patient satisfaction and the overall effectiveness of interventions [41]. From a broader social perspective, strengthening community participation, improving social functioning, and enhancing quality of life can help reduce relapse rates, hospital readmissions, and related healthcare costs [26, 36]. As a result, the integration of such activities into treatment and rehabilitation programs is strongly recommended. these interventions, as a complement to pharmacological treatment, can play a vital role in improving quality of life, promoting overall health, and facilitating successful reintegration into society [32, 34]. Despite evidence of the positive effect of physical activity on improving the health of people with schizophrenia, most previous studies have focused on individual or aerobic programs, or have been conducted cross-sectionally or non-Interventionally, and have not examined the direct and causal effect of structured group programs on various dimensions of quality of life [29]. Furthermore, most of these studies have been conducted in Western countries and in general or mixed populations (including men and women), while the effect of group sports-recreational interventions in different cultural contexts, especially among male patients with chronic schizophrenia in rehabilitation centers, has been less studied. For instance, Takeda et al. [39] found a significant positive association between group-based recreational sports and mental health among men in Japan, while this relationship was not observed among women [39]. Similarly, Ngamaba et al. [29] reported that individuals with schizophrenia who participate more frequently in sports and recreational activities exhibit higher quality of life scores [29]. Nevertheless, consensus on the most effective type of physical activity for this population remains limited, highlighting the need for further investigation. Therefore, the present study was designed to fill this knowledge gap by implementing a 12-week structured group sports-recreational activity program and examining its effect on various dimensions of quality of life in male patients with schizophrenia in southern Iran. This study can help improve the design of non-pharmacological interventions in the field of mental health by providing empirical evidence from a less studied population.

## Methods

### Study design

This semi-experimental study was conducted to investigate the effect of participation in group sports-recreational activities on the quality of life of male patients with schizophrenia. This study was designed, conducted, and reported in accordance with the principles and checklist of the CONSORT 2010 reporting guidelines for semi-experimental studies. The steps including sample selection, random allocation, intervention, and data analysis were organized according to the structure proposed by this guideline to ensure transparency, accuracy, and reproducibility of the results. Quality of life scores at baseline and after the intervention (post-test) were analyzed using analysis of covariance (ANCOVA).

### Patients and procedures

The statistical population of the present study included all male patients with schizophrenia (n = 75) who were hospitalized in 2024 at Hakim Neuropsychiatric Rehabilitation Center in Susa (a city in southwestern Iran). From these individuals, sample selection was performed based on the study’s inclusion and exclusion criteria. In the first stage, patients were screened for inclusion criteria including a definitive diagnosis of schizophrenia by a psychiatrist based on DSM-V, age between 18 and 65 years, ability to participate in physical activities, and no substance dependence or physical disability. After screening, 60 eligible patients were identified and randomly assigned to a experimental (n = 30) and a control (n = 30) group. Fifteen subjects were excluded from the study for the following reasons: eight subjects did not meet the inclusion criteria (including no definitive DSM-V diagnosis, substance dependence, or physical limitation), four subjects were unwilling to participate in the recreational sports program, and three subjects were excluded due to absence from intervention sessions or the post-test. Finally, 60 eligible patients were randomly assigned to the intervention and control groups in a 1:1 ratio. The randomization process was performed after screening to minimize the possibility of selection bias. All participants were referred by psychiatrists, nurses, occupational therapists, clinical psychologists, or volunteers. only male patients participated in this study because the research was conducted in a psychiatric rehabilitation center for men and access to female patients was not possible due to cultural and institutional constraints. This selection was also made to maintain sample homogeneity and control for gender-related confounding factors. The study was conducted in accordance with the guidelines of the Declaration of Helsinki for human research. Informed consent was obtained from participants after detailed explanations of the aims and benefits of the study. This study was approved by the Research Institute of Movement Sciences, Kharazmi University (IR-KHU.KRC.1000.227). The clinical status of all participants was confirmed by licensed psychiatrists and based on the criteria of the Diagnostic and Statistical Manual of Mental Disorders, Fifth Edition (DSM-V). Only patients with a stable clinical status and without acute symptoms of psychosis, suicidal ideation, or severe behavioral disorders were included in the study. The patients’ medication stability was confirmed by reviewing medical records and consulting with the psychiatrist and ward nurses to maintain homogeneity between groups.

Inclusion criteria were as follows: (a) a diagnosis of schizophrenia by a psychiatrist based on DSM-V diagnostic criteria, (b) age between 18 and 65 years, (c) having at least a primary education, (d) no substance dependence, (e) no significant illness or physical disability that limited participation in physical activities, and (f) being male. Patients who missed more than three sessions, patients who suffered from mental retardation (either based on medical history and a score below 70 on the Wechsler Adult Intelligence Scale (WAIS)), or showed symptoms of (a) psychosis, (b) delusions, (c) widespread suicidal ideation, or (d) aggressive behavior were excluded from the study. The Mini-Mental State Examination (MMSE) was used to assess cognitive status, and patients with an MMSE score of less than 24 were identified as having severe cognitive impairment [45]. A research associate explained the purpose of the study and described individual rights and potential risks to each patient before obtaining written consent. After the sampling process was completed, all eligible patients were randomly assigned in a 1:1 ratio to either the intervention group (n = 30) or the control group (n = 30). Randomization was performed using a computer-generated random identification number. All patients maintained their initial antipsychotic treatments. Patients with insomnia or anxiety were treated temporarily with benzodiazepines. Patients experiencing extrapyramidal symptoms were treated briefly with trihexyphenidyl.

In this semi-experimental study, assessments were conducted in two phases, including pre-test (at the beginning of the study) and post-test (at the end of the 12th week). In the pre-test phase, quality of life components including physical health, mental health, social relationships, environmental health, and overall quality of life were assessed. After the 12-week intervention, the same assessments were conducted again in the post-test phase to examine possible changes. Data collection was conducted by a trained research assistant who was responsible for conducting interviews and helping participants complete self-report questionnaires. To maintain the accuracy and consistency of the assessments, two assessors were trained by experienced clinical psychologists, and prior to the final implementation of the assessments, necessary coordination was made between the assessors and the principal investigator to ensure uniformity of scoring and data interpretation. All participants were recruited from the rehabilitation wards of Hakim Psychiatric Center and, prior to entering the study, participated in the center’s standard rehabilitation programs, including health education sessions, nutritional counseling, nursing care, and low-dose drug treatment. In addition to these standard activities, the experimental group participated in a group sports-recreational program. Participants in the control group continued the psychiatric center’s usual rehabilitation program, which included medication management, health education sessions, occupational therapy, and group discussions supervised by the clinical team. This program placed both groups in similar conditions in terms of the amount of social interaction, therapeutic contact, and attention from the treatment staff. However, the control group did not participate in any structured sports or recreational activities. This design allowed for the examination of the additional effect of the group sports-recreational program compared to usual rehabilitation care.

### Intervention

#### Group sports-recreational activities

The 12-week intervention was designed as a multi-segment, structured group program combining physical and recreational elements in accordance with the European Psychiatric Association (EPA) guidelines for physical activity in severe mental disorders [13, 24, 38]. The program was organized into three progressive phases, as detailed in Table 1.

**Table 1:** Structure of the 12-week group sports-recreational program

Phase	Weeks	Focus	Example activities	Session structure
Phase 1	1–4	Light aerobic and stretching exercises aimed at improving body awareness and motor readiness	Stretching, rhythmic movements with music	5-min warm-up, 10-min main activity, 5-min cool-down
Phase 2	5–8	Moderate-intensity group activities to increase endurance, coordination, and cognitive-social engagement	Walking, jogging, darts, table tennis	5-min warm-up, 10-min main activity, 5-min cool-down
Phase 3	9–12	Group games and outdoor recreation sessions to promote social interaction, teamwork, and a sense of belonging	Soccer, volleyball, outdoor recreation	5-min warm-up, 10-min main activity, 5-min cool-down

All sessions lasted approximately 20 min and were conducted three times per week, with 1 day of rest between sessions. Activities took place indoors or, weather permitting, in the hospital yard. Each session was supervised by a physical education specialist (the researcher) and a physiotherapist assistant to ensure safety, consistency, and a balance between physical activity and psychosocial stimulation. A physician examined the participants before each session and was present during the activities. Sessions were scheduled for 10:00 AM, 1 h after breakfast. After each session, participants were provided with a drink.

### Tools

#### Demographic information questionnaire

In this study, personal information of individuals, including age, body mass index, level of education, marital status, hospital visit frequency, duration of illness, and the amount of specific medication usage, was reported.

#### Quality of life questionnaire

The World Health Organization Quality of Life (WHOQOL) questionnaire is a self-report tool designed by the World Health Organization in 1996 for assessing the quality of life of individuals. The short form of the questionnaire consists of 26 items derived from the original 100-item questionnaire and assesses quality of life in four domains: physical health (seven questions), psychological health (six questions), social relationships (three questions), and environmental health (eight questions). Respondents answer each question on a 5-point Likert scale. Reverse scoring is applied to questions 3, 4, and 26. Following the necessary calculations within each domain, a score ranging from 4 to 20 is obtained for each domain, where a score of 4 represents the poorest condition and a score of 20 indicates the best condition in the respective domain. These scores can be converted to a scale of 0–100. This scale has been translated into 19 different languages and is used in various countries to measure individuals’ quality of life. The results indicate acceptable validity and reliability for this scale [22, 35]. The World Health Organization research group considers this scale to be a cross-cultural instrument, which is why it is used in various cultures [30]. The Iranian version of this questionnaire was standardized by Najat et al. (2006), and the Cronbach’s alpha coefficients for the domains of physical health, psychological health, social relationships, and environmental health were reported as 0.87, 0.74, 0.55, and 0.74, respectively [28]. In the current study, the Cronbach’s alpha coefficient for the entire test was calculated to be 0.87.

### Sample size and power

The sample size in this study was determined based on the previous studies [19]. Statistical power calculations were performed using G*Power version 3.1 software. Considering a medium effect size (Effect Size = 0.25), a significance level of 0.05 and a test power of 0.80 [12], the minimum sample size required for analysis of covariance (ANCOVA) was estimated to be 52 participants (26 per group). To compensate for possible participant dropout, the sample size was increased to 60 participants (30 per group).

### Data analysis

For the evaluation of quality-of-life components, analysis of covariance (ANCOVA) was employed, using pretest values as covariates and posttest values as dependent variables [40, 42]. Prior to data analysis, all assumptions of ANCOVA, including normality, homogeneity of regression slopes, and linearity, were assessed and met. SPSS software was utilized for all statistical analyses, and a significance level of *p* < 0.05 was set.

## Results

### Demographic and basic information

Out of the 75 subjects evaluated, 60 patients were eventually randomly assigned to the intervention and control groups (30 each). All participants completed the 12-week study period without dropping out. Patients maintained their previous antipsychotic medication regimen unchanged during the study (including clozapine, olanzapine, quetiapine, aripiprazole, ziprasidone, risperidone, amisulpride, and paliperidone). In total, 42 patients were treated with only one antipsychotic medication and 18 patients with two antipsychotics; 13 patients also received benzodiazepines and 11 patients received tricyclic antidepressants. The age range of the patients was between 18 and 65 years, and there were no significant differences in demographic and clinical data (age, body mass index, marital status, education, family history, frequency of hospitalization, duration of illness) between the intervention and control groups at baseline (all *p* > 0.05). In addition, the doses of antipsychotic medications used by patients were converted to equivalents of olanzapine [21], and there were no significant differences in olanzapine equivalent doses between the two groups (*p* > 0.05). There were no significant(all *p* > 0.05) differences in the use of benzodiazepines and tricyclic antidepressants between the two groups (see Table 2).

**Table 2:** Demographic and clinical data of the experimental and control groups at baseline

	Experimental (n =)	Control(n =)	F(p)
Age (years)	53.45 ± 5.78	54.10 ± 3.70	14(0.78)
BMI	29.44 ± 2.79	29.80 ± 2.46	19(0.67)
Marital status (married/unmarried/divorce or widowhood)	20/4/6	17/5/8	2.78(0.22)
Education (years)	8.65 ± 2.10	8.60 ± 2.11	0.10(0.95)
Family history (no/yes)	21/19	19/11	1.42(0.39)
Frequency of hospitalization (frequency)	3.89 ± 2.70	3.92 ± 2.73	− 0.32(0.99)
Illness duration (years)	31.50 ± 8.62	31.75 ± 9.60	0.17(0.73)
Drug dose of equivalent OLZ (mg/d)	12.89 ± 5.56	13.10 ± 5.50	0.36(0.49)
Benzodiazepines (no/yes)	22/8	20/10	0.06(0.98)
Trihexyphenidyl (no/yes)	20/10	23/7	0.29(0.59)

BMI, body mass index; OLZ, olanzapine

### The effect of sports-recreational group activities on quality of life

After collecting the data, preliminary assumptions for the analysis of covariance (ANCOVA) and one-way and two-way analysis of variance (ANOVA) were examined using the Kolmogorov‒Smirnov test, the homogeneity of variances was assessed using Levene’s test, and variance‒covariance matrices were examined using Box’s *M* test. The results indicated that the assumptions for ANCOVA and ANOVA were met (all *p* > 0.05). All variables in the intervention group showed statistically significant improvement from baseline to post-intervention. The raw data for the groups are presented in Table 3.

**Table 3:** Outcome measures at pre intervention and post intervention for the experimental and control groups

Variables	Pre intervention Post intervention					Mean difference (post intervention)	*P* value
	Experimental mean (SD)	Control mean (SD)	Experimental mean (SD)		Control mean (SD)		
Physical health	14.20(2.11)	14.29(2.40)	19.10(1.84)	14.31 (2.10)		4.90	0.002*
Mental health	14.60(1.76)	15.20(3.47)	23.53(1.60)	15.10(2.99)		8.93	0.001*
Community Relations	12.6(1.36)	34.6(1.22)	9.43(1.50)	6.35(1.27)		3.31	0.002*
Environmental health	23.64(2.03)	24.12(1.64)	27.20(2.02)	24.36(1.97)		3.56	0.003*
Quality of life	58.56(1.81)	59.95(2.18)	79.26(1.74)	60.12(2.08)		20.7	0.001*

*Level of significance *P* < 0.05

**Fig. 1:**
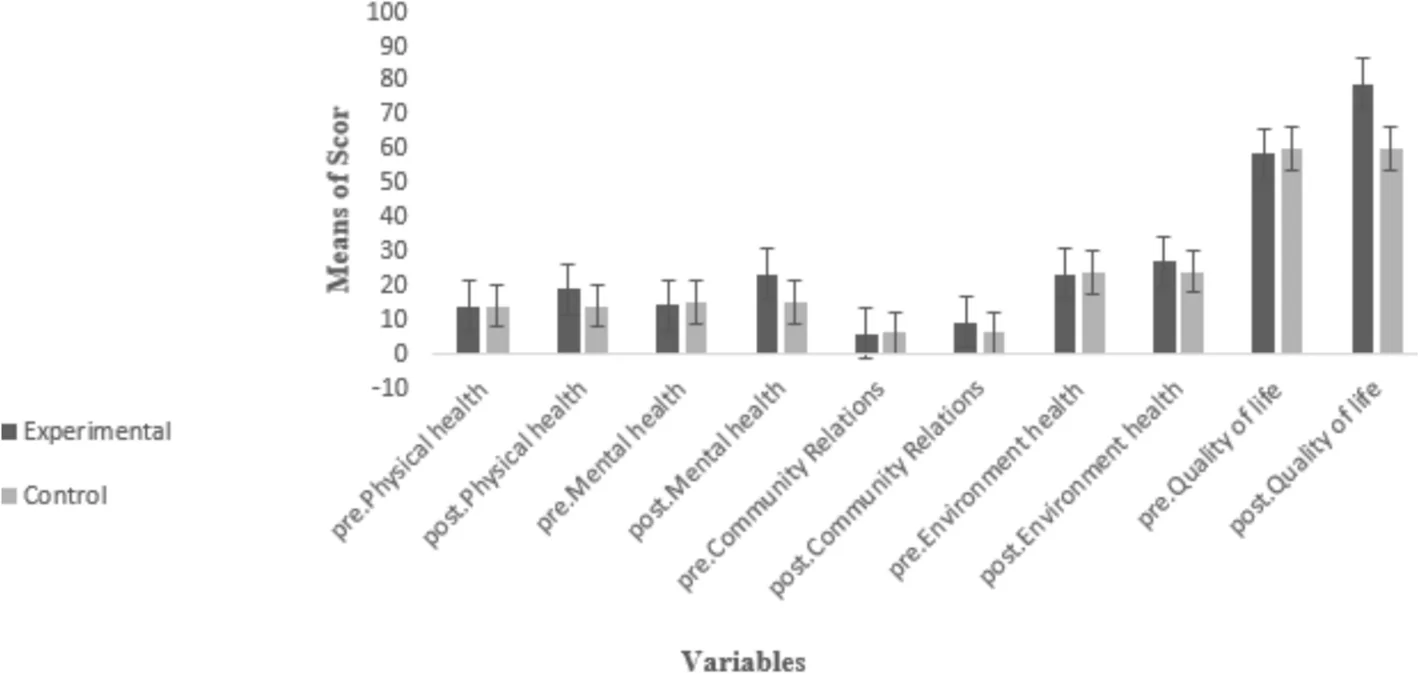
Performances on subscales of quality of life in the pretest and posttest

The results of the ANCOVA test indicate that group sports-recreational activities have a statistically significant impact on the physical health component (*F*(1,58) = 105.413, *p* = 0.001, *η*^2^ = 0.79). Furthermore, the results between the groups show that the experimental group had a greater improvement in the physical health component than the control group (*F*(1,58) = 12.26, *p* = 0.002, *η*^2^ = 0.30). the results of ANCOVA also demonstrated that group sports-recreational activities had a significant effect on the psychological health (*F*(1,58) = 197.22, *p* = 0.001, *η*^2^ = 0.87), social relationships (*F*(1,58) = 95.66, *p* = 0.002, *η*^2^ = 0.76), environmental health (F(1,58) = 88.90, *p* = 0.002, *η*^2^ = 0.65) and overall quality of life (*F*(1,58) = 243.89, *p* = 0.001, *η*^2^ = 0.89) scores of patients with schizophrenia. Additionally, based on the between-group results, the experimental group showed greater improvement in the psychological health (*F*(1,58) = 31.22, *p* = 0.001, *η*^2^ = 0.52), social relationships)F(1,58) = 12.20, *P* = 0.002, η^2^ = 0.37(, environmental health (*F*(1,58) = 10.80, *p* = 0.003, *η*^2^ = 0.30) and overall quality of life (*F*(1,58) = 45.42, *p* = 0.001, *η*^2^ = 0.61) components compared to the control group. Figure 1 represents the differences between the pretest and posttest stages for the experimental and control groups.

## Discussion

This study aimed to investigate the effects of a 12-week group sport-recreational activity program on the quality of life of male patients with schizophrenia. The results showed that participation in such activities had a positive and significant effect on overall quality of life, with improvements observed in all domains of the WHOQOL-BREF questionnaire—including physical health, mental health, social relationships, and environmental health. The intervention group showed significant improvement compared to the control group, highlighting the therapeutic value of structured group sport activities in the management of schizophrenia. Traditional approaches to the treatment of schizophrenia, including pharmacological and psychotherapy, focus primarily on psychological symptoms but often fail to address the physical and functional disabilities associated with the disorder [8, 33]. In contrast, physical activity has the unique ability to simultaneously improve both physical and mental health [1, 19]. The findings of this study are in line with several other studies that have demonstrated the beneficial effects of physical activities on improving the quality of life of individuals with schizophrenia [9, 19, 29]. However, factors such as the type and duration of exercise, participants’ gender, the nature of the illness, sample size, and the timing of variable measurements postintervention may introduce differences in the outcomes [8].

In the area of physical health, the intervention resulted in improvements in energy levels, functional capacity, and the ability to perform daily activities. Since antipsychotic medications, especially second-generation drugs, often cause weight gain and metabolic problems that reduce quality of life [44], participation in physical activity can be a valuable strategy to counteract these effects. Exercise can produce weight loss at a level similar to structured weight management programs, increase energy levels and functional independence, and reduce fatigue [13, 32]. These results are consistent with previous studies that have shown that regular physical activity reduces obesity and improves physical function in patients with schizophrenia [3, 13]. Although some studies have found no significant improvements in physical health [11, 16], these differences are likely due to differences in sample characteristics or measurement instruments.

Regarding psychological health, participation in group sports and recreational activities increased participants’ self-esteem, motivation, and emotional well-being. The improvements in psychological health likely reflected increased emotional stability, reduced stress, and feelings of satisfaction resulting from participation in regular, enjoyable group activities. Although constructs such as motivation and self-efficacy were not directly measured in this study, previous studies have shown that group activities can indirectly enhance these psychological processes and lead to improved subjective well-being and greater adherence to exercise programs [13, 25]. Motivation plays a key role in maintaining lifestyle changes and reducing symptoms of depression, anxiety, and hopelessness—issues that are common in schizophrenia [5]. Physical activity helps patients move from passive to active participation in their recovery by enhancing self-efficacy and personal agency [25, 34]. Furthermore, the group nature of the intervention increased social connections, reduced isolation, strengthened interpersonal relationships, and developed self-esteem. These findings are consistent with previous research that has shown that structured exercise increases psychological resilience and improves cognitive [4, 8, 17]. Physical activity may also regulate neurotransmitter systems—including dopamine, serotonin, and glutamate—and thereby improve mood and cognitive function [13, 19]. The present findings are consistent with the results of some studies [19, 29], but also inconsistent with some studies [37], which may be due to differences in study design and measurement instruments.

Regarding social relationships, the intervention significantly improved patients’ satisfaction with personal relationships, social support, and communication skills. Social participation is a key factor in the improvement and satisfaction with life in patients with schizophrenia [20]. Participating in group activities gives patients the opportunity to develop their social skills, express their emotions, and experience a sense of belonging [32]. Recreational sports are not only a form of rehabilitation but also provide a platform for expressing emotions and social rehabilitation [14, 19]. The results of this study are consistent with previous studies that emphasize the role of group activities in promoting social bonding and reducing isolation [15, 29]. The use of exercise programs alongside medication and psychotherapy can accelerate the improvement of social functioning and increase quality of life [13]. Differences in findings with some studies [6] are likely due to differences in the characteristics of the population studied or the instruments used.

Improvements in environmental health—including feelings of safety, comfort, and access to recreational facilities—suggest that the supportive and collaborative environment of the program implementation likely played a role in increasing patient satisfaction and engagement. Feeling accepted and respected within a social group can have anti-stigma effects, a major barrier to recovery and predictor of poor mental quality of life [6, 25]. Although the social stigma variable was not directly measured in the present study, based on previous evidence, community-based group interventions can be effective in reducing feelings of stigma and social isolation by increasing patients’ social participation and their presence in positive group situations [20]. Consequently, the environmental context is a key determinant of the success of interventions,spaces that foster a sense of comfort, inclusion, and access can improve treatment outcomes [7, 20]. The findings of this study are consistent with the evidence of some previous studies [19, 29], while differences with some other studies [6, 18] are likely related to differences in study context and assessment tools.

Overall, the results of this study suggest that integrating structured, enjoyable, and social physical activities into treatment programs can significantly improve the quality of life of patients with schizophrenia. In addition to improving physical and mental health, these interventions promote social inclusion and reduce stigma, and simultaneously improve various dimensions of well-being. In the future, it is necessary to examine the long-term sustainability of these effects and determine the optimal type, intensity, and duration of physical activities for different patient groups. In addition, integrating exercise programs into community mental health services can improve access and continuity of care.

### Limitation

The present study had several limitations. First, the study included only male patients, which significantly limits the generalizability of the findings to female patients with schizophrenia. Given that schizophrenia may present differently across genders—including differences in symptom severity, treatment response, and social functioning—the effectiveness of group sports-recreational activities may also differ between men and women. Therefore, caution is warranted when applying these findings to female populations. Future research should examine similar interventions among female and mixed-gender populations to increase the external validity of the results and to identify possible gender differences in treatment response. It should also be noted that the psychological and social status of respondents during the completion of the questionnaire and exercises can affect the quality of their responses and performance. Future studies should increase the sample size and provide a richer and more comprehensive understanding of the physiological variables that mediate the effectiveness of combined psychoeducational and pharmacological treatments for psychiatric disorders. In addition, future studies should extend the duration of intervention programs and examine whether the benefits are sustained over a longer period of time.

## Conclusion

This study showed that implementing a group program of sports-recreational activities for patients with schizophrenia is feasible and effective. The results indicated significant improvements in physical, mental, social, and environmental health, and consequently improved the patients’ quality of life. Accordingly, combining pharmacological treatments with innovative behavioral interventions such as group sports activities can be beneficial as a complementary approach in the recovery and rehabilitation of patients. However, attention should be paid to the potential negative consequences of sports activities, including physical injuries, psychological pressures from competition, and the risk of addictive behaviors. Also, factors such as social anxiety, impaired emotional perception, and negative past experiences may prevent patients from participating in these activities. Therefore, it is recommended that mental health professionals carefully consider the individual characteristics and preferences of patients when designing and implementing such programs.

## Data Availability

The datasets generated during the current study are available from the corresponding author on reasonable request.
